# A Deep Learning-Based Approach for the Detection of Early Signs of Gingivitis in Orthodontic Patients Using Faster Region-Based Convolutional Neural Networks

**DOI:** 10.3390/ijerph17228447

**Published:** 2020-11-15

**Authors:** Dima M. Alalharith, Hajar M. Alharthi, Wejdan M. Alghamdi, Yasmine M. Alsenbel, Nida Aslam, Irfan Ullah Khan, Suliman Y. Shahin, Simona Dianišková, Muhanad S. Alhareky, Kasumi K. Barouch

**Affiliations:** 1 Department of Computer Science, College of Computer Science and Information Technology, Imam Abdulrahman Bin Faisal University, Dammam 31441, Saudi Arabia; 2160000040@iau.edu.sa (D.M.A.); 2160006145@iau.edu.sa (H.M.A.); 2160000951@iau.edu.sa (W.M.A.); 2160001619@iau.edu.sa (Y.M.A.); naslam@iau.edu.sa (N.A.); 2 Division of Orthodontics, Department of Preventive Dental Science, College of Dentistry, Imam Abdulrahman Bin Faisal University, Dammam 31441, Saudi Arabia; sshahin@iau.edu.sa; 3 Department of Orthodontics, The Slovak Medical University, 833 03 Bratislava, Slovakia; simonadianiskova@gmail.com; 4 Division of Pediatric Dentistry, Department of Preventive Dental Science, College of Dentistry, Imam Abdulrahman Bin Faisal University, Dammam 31441, Saudi Arabia; malhareky@iau.edu.sa; 5 Division of Periodontology, Department of Preventive Dental Science, College of Dentistry, Imam Abdulrahman Bin Faisal University, Dammam 31441, Saudi Arabia; kasumibrch@gmail.com

**Keywords:** gingivitis, periodontal disease, deep learning, convolutional neural networks

## Abstract

Computer-based technologies play a central role in the dentistry field, as they present many methods for diagnosing and detecting various diseases, such as periodontitis. The current study aimed to develop and evaluate the state-of-the-art object detection and recognition techniques and deep learning algorithms for the automatic detection of periodontal disease in orthodontic patients using intraoral images. In this study, a total of 134 intraoral images were divided into a training dataset (*n* = 107 [80%]) and a test dataset (*n* = 27 [20%]). Two Faster Region-based Convolutional Neural Network (R-CNN) models using ResNet-50 Convolutional Neural Network (CNN) were developed. The first model detects the teeth to locate the region of interest (ROI), while the second model detects gingival inflammation. The detection accuracy, precision, recall, and mean average precision (mAP) were calculated to verify the significance of the proposed model. The teeth detection model achieved an accuracy, precision, recall, and mAP of 100 %, 100%, 51.85%, and 100%, respectively. The inflammation detection model achieved an accuracy, precision, recall, and mAP of 77.12%, 88.02%, 41.75%, and 68.19%, respectively. This study proved the viability of deep learning models for the detection and diagnosis of gingivitis in intraoral images. Hence, this highlights its potential usability in the field of dentistry and aiding in reducing the severity of periodontal disease globally through preemptive non-invasive diagnosis.

## 1. Introduction

Periodontal diseases are a group of oral inflammations that affect gum tissue and the supporting structures of the teeth. Gingivitis is the first and mildest stage of progression of periodontal disease, which is non-destructive of bone in nature and reversible if preemptively diagnosed [1]. When left untreated, however, gingivitis may potentially lead to a case of periodontitis, eventually leading to loss of periodontal ligament and the destruction of the surrounding bone structures [2].

Periodontal disease is deemed to be the leading cause of tooth loss worldwide as it is highly prevalent in both developed and underdeveloped countries, affecting about 20–50% of the global population [3]. In addition, several epidemiological studies report that periodontal disease is linked to other serious health conditions such as cancer, cardiovascular disease, and Type-II diabetes [3,4,5].

Clinical diagnosis methods of gingivitis and chronic periodontitis include measuring the periodontal probing depth (PPD), bleeding on probing, and radiographic assessment of alveolar bone loss [6]. However, these methods are invasive and often painful for patients, and the measurements can differ between examiners using different probes, even for repeated site measurements [7,8]. Hence, several newer generation probes have been developed to improve accuracy when measuring the periodontal probing depth (PPD) [9]. However, these measurements can be performed accurately by trained dental specialists only, which highlights the need for a more accessible and non-invasive diagnosis method [10]. Patients seeking orthodontic treatment require careful monitoring of their gingival condition as orthodontic appliances can cause transient gingivitis due to challenges in practicing proper oral hygiene measures [11]. Though orthodontic treatment in patients with a previous history of cured periodontal disease and gingivitis is possible, caution must be exercised to avoid further periodontal breakdown and careful monitoring is essential during treatment [12]. Thus, image analysis can play a pivotal role in monitoring the gingival condition of orthodontic patients.

Throughout the medical field, the use of machine learning and deep learning techniques has rapidly become more successful in the construction of automated diagnostic systems that diagnose various diseases. In particular, studies have demonstrated that convolutional neural networks (CNNs),which are used in computer vision tasks such as object detection and recognition, can be utilized successfully for the detection and diagnosis of breast and thyroid cancer, ulcerative colitis, and chronic obstructive pulmonary disease such as pneumonia and tuberculosis [13,14,15].

Similarly, in the dental field, research shows that the detection and diagnosis of periodontal disease using machine learning techniques has also been proven to be successful. However, the use of data such as microscopic images of plaque [16], radiographic images [17], and fluorescent images [18] in the majority of these studies limits their applications to a strictly clinical setting. In contrast, studies that are purely based on intraoral images are limited and focus on aggressive cases of periodontitis [19] rather than preemptively diagnosing the disease in its earliest stage, i.e., gingivitis. Therefore, the aim of the current study was to improve upon previous studies and evaluate the effectiveness of deep learning-based CNNs for the preemptive detection and diagnosis of periodontal disease and gingivitis by using intraoral images.

## 2. Materials and Methods

### 2.1. Dataset and Ground Truth Annotations

The study was conducted in the Department of Computer Science and the Department of Preventative Dental Science of Imam Abdulrahman bin Faisal University and was approved by the Deanship of Scientific Research of Imam Abdulrahman bin Faisal University (IRB No. 2018-02-285). The intraoral image dataset was acquired on 7 October 2019; it was obtained from the College of Dentistry (Imam Abdulrahman bin Faisal University) along with the clinical findings of the maxillary central incisors’ gingiva in each image.

The dataset consists of 47 male and female orthodontic patients. It includes patients of different age groups, smokers, pregnant and lactating women, and patients undergoing the treatment of systemic disease with a stable condition and excludes any patients with active systemic disease and undergoing cancer treatment.

After obtaining the consent of all 47 patients, an intraoral image of each patient was taken at three time-points (T0 = before orthodontic treatment, T1 = one week after orthodontic treatment, T2 = four weeks after orthodontic treatment), thus resulting in a total of 141 images. After removing seven images due to duplication and incorrect clinical scores, a total of 134 remained. In each image, the gingiva of the maxillary central incisors was annotated at the distal, middle, and mesial regions, resulting in a total of 6 regions for each image, i.e., 804 regions in total for all 134 images. Each region was examined by expert dentists and labeled as either inflamed (*n* = 305 [37.9%]) or non-inflamed (*n* = 499 [62.1%]) using the gingival index described by Löe and Silness [20] as the diagnostic criteria. For anonymity, the intraoral image dataset was used without the extraction of patients’ personal information such as name, age, gender, and address.

### 2.2. Pre-Processing

The dataset was divided into training and testing with a ratio of 80:20. Therefore, 107 intraoral images were used in training the model, and 27 images were used for testing. The data were split based on patients, i.e., all time-points belonging to a single patient are placed in a single set, either training or testing. This was conducted to ensure that no data are shared between the training and testing sets, hence avoiding data leakage and overfitting.

### 2.3. Faster R-CNN

Faster Region-based Convolutional Neural Network (Faster R-CNN) [21] is a CNN-based algorithm that aims at detecting and classifying regions of interest (ROIs) in an input image. Faster R-CNN comprises two main components: a region proposal network (RPN), which intelligently proposes regions of interest, and a convolutional neural network that classifies the objects in these regions.

**Figure 1:**
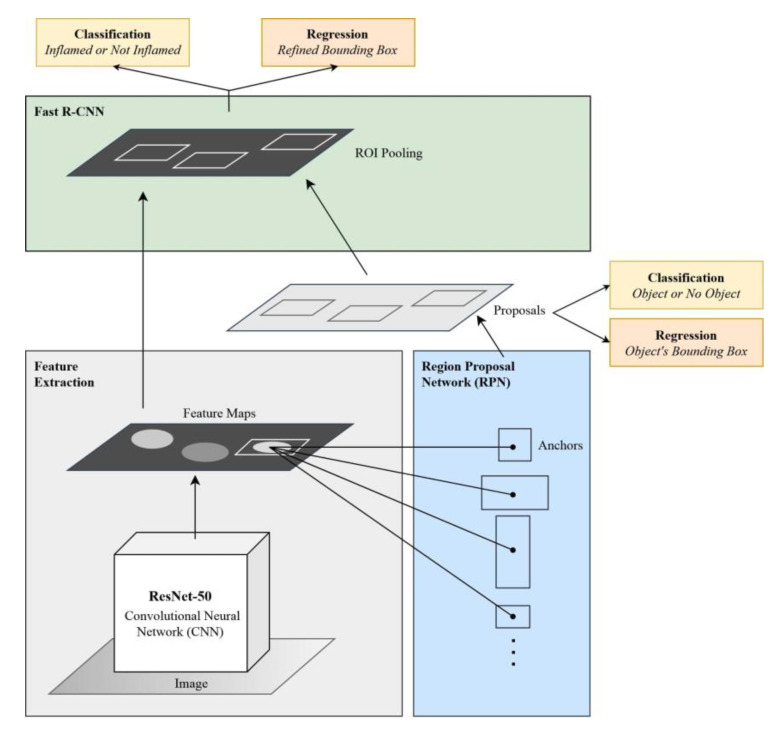
Architecture of the Faster R-CNN model.

In Faster R-CNN, the input image is passed into a feature extractor, which is a CNN that produces feature maps and passes them to the RPN. For the current study, ResNet-50 CNN was used as a feature extractor. Next, the RPN uses a sliding window over the feature maps and produces *n* anchor boxes in varying shapes and sizes. Using classification and regression, the RPN predicts the likelihood that a single anchor contains an object and calculates its bounding box. Since the ROIs are of varying sizes, they must be passed to an ROI pooling layer so that the proposals are converted into fixed-sized shapes to ensure compatibility with the fully connected layer. Finally, each finalized proposal is passed into a sequence of fully connected layers, which branch into two output layers: the first is a SoftMax layer that predicts the class of the object, while the second calculates the object’s refined bounding box. The architecture of Faster R-CNN is shown in Figure 1.

### 2.4. Teeth Detection Model and Cropping Procedure

Using TensorFlow’s Object Detection API, the Faster R-CNN object detection model was constructed for the purpose of detecting teeth using ResNet-50 CNN as a feature extractor. This process was performed on a virtual Quadro P4000 GPU with an 8 GB RAM and an Ubuntu 18.04 operating system (Canonical Ltd., Tokyo, Japan). The input images were resized into 500 × 500 pixels while maintaining their aspect ratio to accommodate the available computational resources. The training parameters were adjusted as follows to achieve a high detection result: a batch size of 1, an initial learning rate of 0.0003, and a batch queue capacity of 50. The TensorFlow Object Detection API, by default, draws the bounding boxes of each detected object. However, visualization utilities were applied and modified to include a custom function that instead returns the four coordinates of the detected bounding box which will be used to crop the ROI.

**Figure 2:**
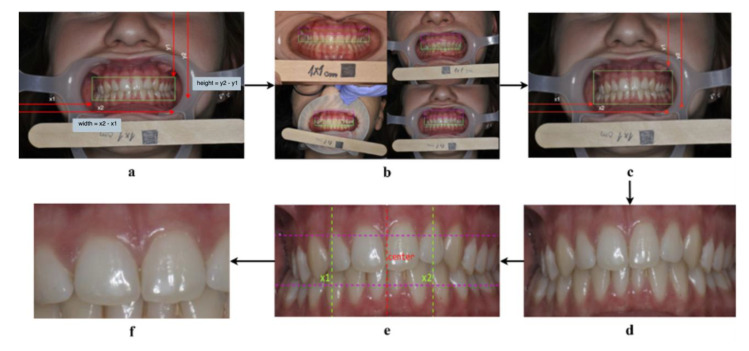
The cropping algorithm steps. (**a**): Computing the height and width of the bounding box. (**b**): Adding k to the upper bound of the bounding box can capture the upper gingiva. (**c**): Expanding the upper and lower bounds of the bounding box. (**d**): A successful capture of the gingival area. (**e**): Narrowing the width of the bounding box to capture the “Big M” region. (**f**): A successful capture of the “Big M” region.

The cropping algorithm (Figure 2) starts by expanding the bounding box to include the gingiva, thus computing y2−y1, which is the height of the bounding box (Figure 2a). Afterwards, it was found that the height of half the bounding box (*k*) can sufficiently capture the gingiva when added to both the upper and lower parts of the box, as shown in Figure 2b. Therefore, the upper bound of the box was expanded by subtracting *k* from the *y*1 coordinate. To expand the lower bound, *k* was added to the *y*2 coordinate (Figure 2c). This results in the successful capture of the gingival area (Figure 2d).

To narrow down the image to the maxillary central incisors (“Big M” region), the width is divided in half to obtain the center point (*q*) of the image i.e., where the “Big M” is assumed to be (see the red dotted line in Figure 2e). Afterwards, each half is divided into two, dividing the image vertically into four quarters, where each quarter is of length *z*. To form the start-point x1, *z* was subtracted from *q*, and added to *q* to form the endpoint x2 (see the green dotted lines in Figure 2e). Finally, the lower third of the image is discarded by dividing the image into thirds and multiplying by two to preserve the “Big M” region (see Figure 2f).

### 2.5. Inflammation Detection Model

**Figure 3:**
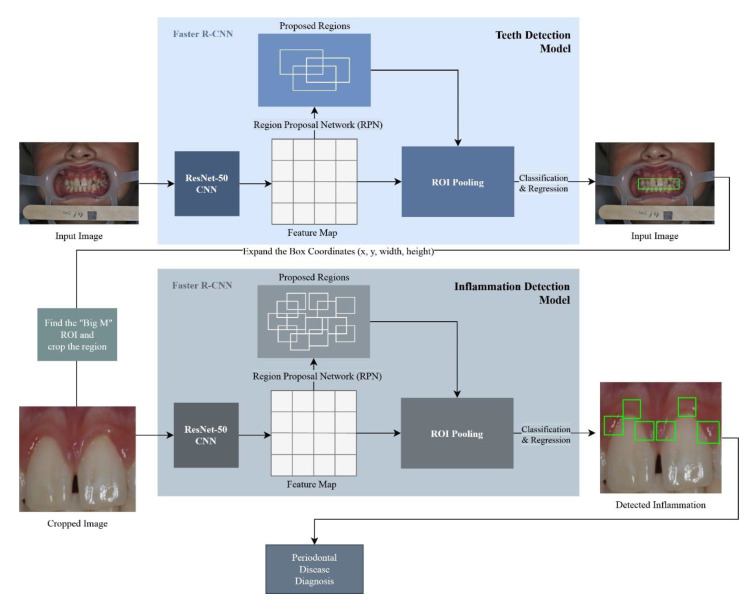
Proposed methodology architecture.

The Faster R-CNN (Region-based Convolutional Neural Network) model was constructed using the TensorFlow v.1.14 Object (Google, Mountain View, CA, USA) Detection API [22]. The Faster R-CNN model was trained on the 107 images produced by the cropping algorithm. The model was trained using two classes: the “inflamed” class, and the “non-inflamed” class. In order to optimize the learning process, the following training parameters were used: a batch size of 1, a learning rate of 0.0003, an Intersection over Union (IoU) threshold of 0.5, and a score threshold of 0.5, in addition to setting the maximum total detections for a single image to 6. Finally, the non-maximum suppression (NMS) algorithm with a SoftMax function was applied to eliminate overlapped detections belonging to the same class. Figure 3 demonstrates an overall view of the proposed approach.

### 2.6. Performance Metrics Used

The mean average precision (mAP), precision, and recall are the most common metrics for the object detection problem. As is true with any other evaluation algorithm, the metrics are evaluated in comparison to the ground truth. In an object detection problem, the ground truth is indicated by the class of the object and its exact location (bounding box) in four coordinates.

**Figure 4:**
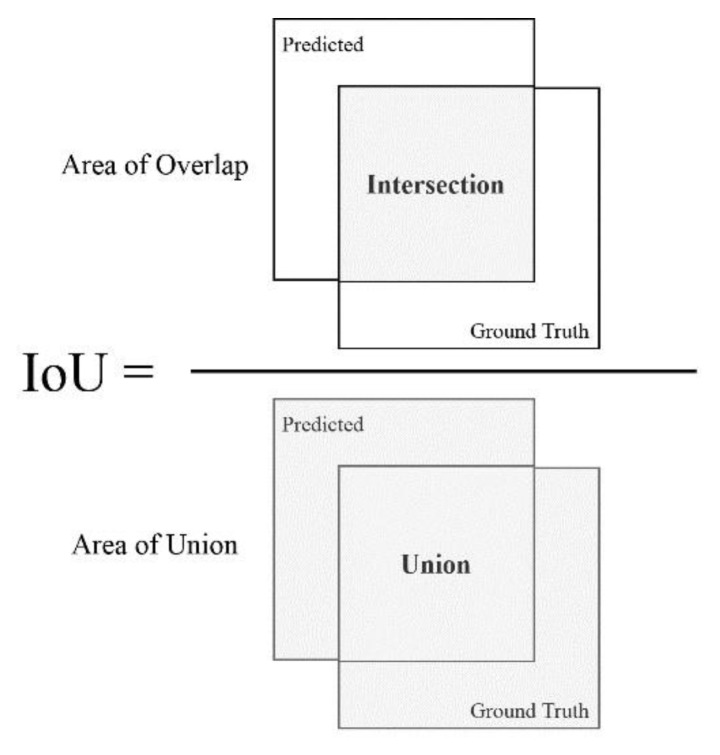
Computing the Intersection over Union (IoU).

In order to calculate the aforementioned metrics, the precision of each detection is compared to the ground truth. The metric that determines such preciseness is the IoU, also referred to as the Jaccard index. The IoU essentially quantifies the amount of overlap between the ground truth and the predicted box (see Figure 4). Using the IoU, a prediction is determined as successful by verifying if the bounding boxes heavily overlap with the ground truth boxes.

To calculate the precision, first the true positives and false positives are determined. A detection is considered a true positive if the IoU > 0.5, and it is considered a false positive if otherwise, where 0.5 is the threshold. This threshold may differ from one object detection problem to another and therefore must be tuned accordingly. Using the true positives and true negatives, the precision is calculated as follows:

(1) $$Precision=\frac{True\;Positives}{True\;Positives+False\;Positives}$$

To calculate the recall, the false negatives are determined, which in an object detection problem is every object our detector has failed to detect. Using true positives and false negatives, the recall is calculated as follows:

(2) $$Recall=\frac{True\;Positives}{True\;Positives+False\;Negatives}$$

Finally, to calculate the mean average precision, the average over the average precisions (APs) of all the classes is computed. As described by Everingham et al. [23], we calculate the mean of the precision values at 11 different confidence thresholds, where the recall at those confidence values ranges from 0 to 1 [0, 0.1, 0.2, …, 0.9, 1.0] using the following equation:

(3) $$AP=\frac{1}{11}\underset{r\;\in \left\{0,\;0.1,\;\ldots ,1\right\}}{\sum }P_{interp\left(r\right)}$$

The precision used in the above equation is interpolated by taking the maximum precision possible at each corresponding recall level *r* using the equation [23], where $p\left(\tilde{r}\right)$ is the precision measured at a recall $\tilde{r}$:

(4) $$P_{interp\left(r\right)}=\underset{\tilde{r}:\;\tilde{r}\geq r}{\max}p\left(\tilde{r}\right)$$

Therefore, the mean average precision (mAP) is the average of all average precisions (APs) of the classes in our data. Nevertheless, depending on class distribution in the data, the average precision may differ from one class to another. Therefore, in object detection problems with a class imbalance, the average precisions for each individual class must be considered when analyzing the model’s results.

In this study, the usage of standardized performance metrics that produce accurate results irrespective of the dataset was quintessential. For this purpose, Padilla and Silva’s [24] implementation was utilized, which compares the ground truths to the model’s detections to evaluate the object detection model accurately and unbiasedly.

## 3. Results

The study was developed using 107 intraoral images for training and 27 intraoral images for testing purposes. Each image was annotated using the provided clinical findings and the assistance of expert dentists.

In this study, the Faster R-CNN-based teeth detection model was used to detect teeth in the intraoral images. This model scores 100% accuracy, 100% precision, 51.85% recall, and 100% mAP (Table 1). Furthermore, since the region of interest is the gingiva of the maxillary central incisors (“Big M” region), a specific algorithm was developed to extract this region after the teeth are detected. The resulting cropped image will be input into the Faster R-CNN inflammation detection model.

**Figure 5:**
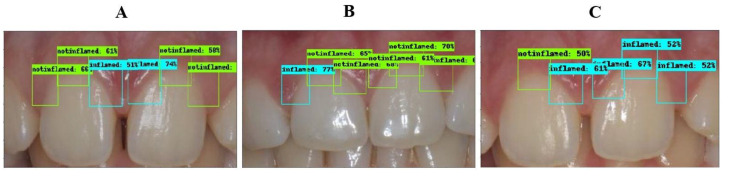
Sample images correctly annotated. (**A**): all six regions are correctly detected. (**B**): distal in tooth No. 11 incorrectly detected as inflamed. (**C**): distal in tooth No. 11 not detected; middle in tooth No. 21 incorrectly detected as inflamed.

The inflammation detection model is responsible for detecting gingival inflammation. It should be known that the ROI is focused on three main regions: the distal, middle, and mesial of the maxillary central incisors. The bounding box of each region was labeled as “inflamed” or “non-inflamed” based on the provided clinical findings. The results of each class are summarized in Table 1. The overall accuracy, precision, recall, and mAP obtained in this model were 77.12%, 88.02%, 41.75%, and 68.19%, respectively. A few sample images annotated by the inflammation detection model are shown in Figure 5 and the corresponding labels and clinical findings can be found in Table 2.

## 4. Discussion

Many patients seeking dental treatment, such as orthodontics, are at risk of developing gingival disease. If patients develop gingivitis, they may be at risk of developing periodontitis when not diagnosed preemptively, which consequently leads to loss of connective tissue, destruction of bone support, and tooth loss. Hence, preemptive detection and diagnosis of gingivitis are the key elements in providing preventive care and treatment for patients suffering from chronic periodontal disease. Using a deep CNN based on the Faster R-CNN architecture, which has been used extensively in the medical field for diagnosing diseases from image data, the model was able to achieve substantial results that contribute to the accurate detection and diagnosis of gingivitis. Hence, this proves the viability of deep learning-based solutions in the dental field.

Needless to say, traditional diagnosis methods are still the most standard practice among professional dentists. Upon evaluation of a patient’s medical history, medication history, and family history of disease as well as extraoral examination, a standard examination of gingival tissues starts with a visual assessment to evaluate the extent of gingival inflammation. Afterwards, periodontal probing is performed by inserting a periodontal probe into the pockets and applying force to measure the periodontal probing depth (PPD) and bleeding on probing (BOD) which indicates a periodontal disease activity. The amount of probing force applied during this procedure can be extremely uncomfortable for the patient and even more so in inflamed tissue as opposed to non-inflamed tissue [6]. Patients with elevated pain may also require the application of local anesthetics during probing if need be [25].

Due to the invasive nature of standard examinations, various efforts have been made towards developing a non-invasive method of detecting and diagnosing periodontal disease and gingivitis, many of which have incorporated machine learning as a result of the rapid advent of computer-aided diagnosis in medicine. In fact, one study using intraoral images of aggressive cases of periodontitis achieved an accuracy and recall of 66.7% and 68.1%, respectively [19]. Unlike traditional machine learning techniques, however, deep CNN algorithms have the capability to efficiently learn representations and extract features that may hold great predictive capabilities due to their deep multi-layer architecture. As a matter of fact, by implementing a deep CNN, our model has achieved an accuracy that is 10% higher than that of models using traditional machine learning methods, thus proving the current technique to be more advantageous than traditional methods.

Furthermore, ResNet, the winning deep learning algorithm of the ImageNet challenge in 2015, has proven to be a groundbreaking innovation by making it possible to train extremely deep neural networks (with up to 152 layers). Before the development of ResNet, this was too complex due to the vanishing gradient problem. ResNet utilizes the concept of skipping connections to skip over certain layers and apply batch normalization in between. This allows the network to reuse activations from previous layers, eliminating the need to recalculate the weights, hence avoiding the vanishing gradient problem and consequently increasing its performance. This influenced us to choose ResNet as a feature extractor for our Faster R-CNN-based inflammation detection model.

As previously mentioned, preemptive diagnosis and treatment of gingivitis are crucial as its progression to periodontitis often leads to irreversible consequences. Due to this, the mean average precision is considered a more important factor in the detection of gingivitis using Faster R-CNN object detection rather than accuracy and/or recall. However, since gingivitis is a mild form of periodontal disease, the subtlety in inflammation may have made it difficult to differentiate between the inflamed and non-inflamed regions. As a result, the study found that the mAP of the non-inflamed class was 21.5% higher than that of the inflamed class, whereas the difference in accuracy was a mere 2.6%. This can of course be overcome by collecting more data from the early stages of gingival inflammation or even using a more powerful CNN as a feature extractor.

Despite the various advantages of this study, several important limitations need to be addressed. The most important limitation is the fact that the study focuses exclusively on the “Big M” region as opposed to the entire gingiva of the anterior region. Another limitation lies in the fact that the sociodemographic characteristics of the study sample were not considered in this study. Furthermore, due to data unavailability, the study was conducted on a total of 134 images which may be considered a small sample size. Additionally, since the model solely distinguishes between inflamed and non-inflamed regions, the study does not delve into differentiating between the different degrees of inflammation. Moreover, it is important to note that assessing the progression of periodontitis can only be performed by examining the degree of alveolar bone loss in radiographs, thus limiting the applications of this study to the diagnosis of gingivitis rather than periodontitis as intraoral images alone cannot be used as a basis for diagnosing periodontitis. However, since the collection and subsequent assessment of radiographs are strictly clinical procedures, intraoral images still serve a valuable purpose for preemptive diagnosis outside of clinical settings, hence offering a multitude of possibilities that surely must be explored. Finally, in future research, more images can be obtained to further validate the model. Experiments could also be conducted to replace the ResNet feature extractor with an even higher performing CNN to explore whether performance can be improved even further. Additionally, further development of deep learning models for the image analysis of the entire oral region can be of further interest to evaluate the validity of using this approach for a thorough diagnosis of the whole gingiva.

## 5. Conclusions

Gingivitis is the first and mildest stage of progression of periodontal disease, which is non-destructive of bone in nature and reversible if preemptively diagnosed. If gingivitis is left untreated and it progresses to periodontitis, it may affect other serious health conditions such as cancer, diabetes, and cardiovascular disease. With such serious consequences, it is important to preemptively diagnose the disease in its early stages to prevent further complications. Findings from our study suggest that providing the field of dentistry with an enhanced non-invasive method to diagnose gingivitis using intraoral images can help to reduce the complications of untreated gingival disease. Aside from the added benefit of it being a cost-effective solution, it also reduces the need for clinical examinations and prevents patients from undergoing costly surgeries to treat the disease in its advanced stages. In future studies, more research is needed in order to include demographic and clinical data to explore the impact of clinical information on the diagnosis of gingivitis.

## Figures and Tables

**Table 1:** Accuracy, precision, recall, and mean average precision (mAP) for both the teeth detection model and inflammation detection model.

		Training *	Testing *	Accuracy	Precision	Recall	mAP
**Teeth Detection Model**	Teeth	107	27	100%	100%	51.85%	100%
**Inflammation Detection Model**	Inflamed	226	79	78.46%	87.14%	35.05%	57.44%
	Non-Inflamed	416	83	75.79%	88.91%	48.47%	78.94%
	Overall Total	642	162	77.12%	88.02%	41.75%	68.19%

* Number of bounding boxes/annotations.

**Table 2:** The results of the inflammation detection model for 3 patients. The detection results match the clinical findings except for the highlighted regions (GT = ground truth).

Patient	Tooth No.11			Tooth No.21		
	Distal	Middle	Mesial	Mesial	Middle	Distal
**A**	Non-Inflamed	Non-Inflamed	Inflamed	Inflamed	Non-Inflamed	Non-Inflamed
**B**	Inflamed (GT: Non-Inflamed)	Non-Inflamed	Non-Inflamed	Non-Inflamed	Non-Inflamed	Non-Inflamed
**C**	Not Detected (GT: Non-Inflamed)	Non-Inflamed	Inflamed	Inflamed	Inflamed (GT: Non-Inflamed)	Inflamed
